# A patient with painful oral ulcers

**DOI:** 10.1016/j.jdcr.2023.10.015

**Published:** 2023-11-03

**Authors:** Patchareya Mekasuwandit, Supapat Laodheerasiri

**Affiliations:** Division of Dermatology, Department of Internal Medicine, Phramongkutklao Hospital, Bangkok, Thailand

**Keywords:** histoplasmosis, lip ulcer, tongue ulcer

## Case presentation

**Fig 1:**
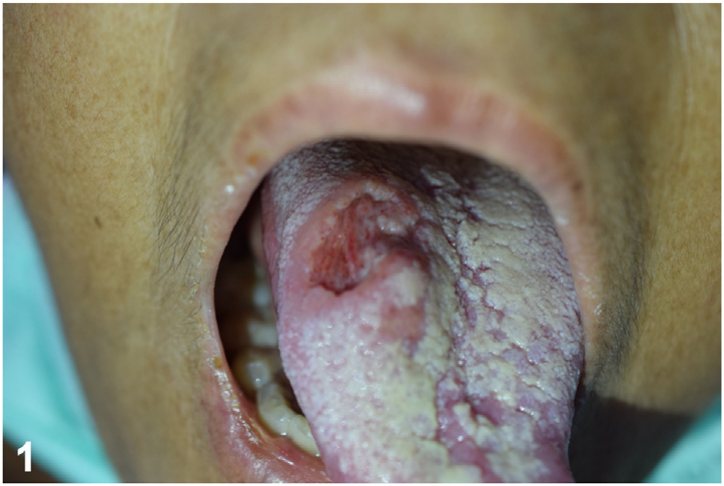

**Fig 2:**
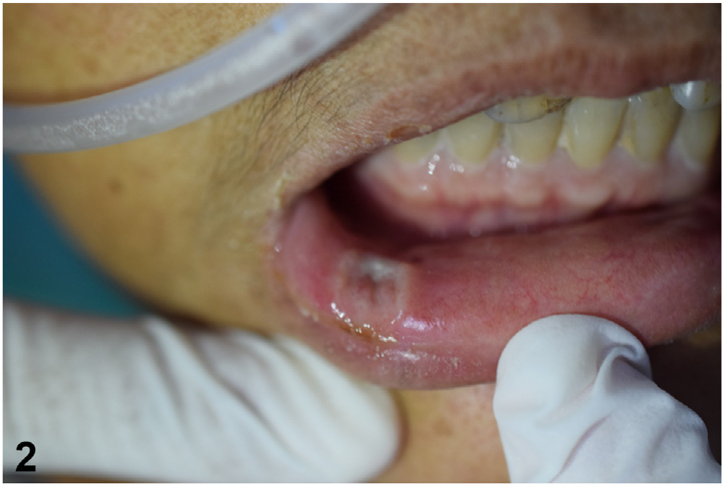

**Fig 3:**
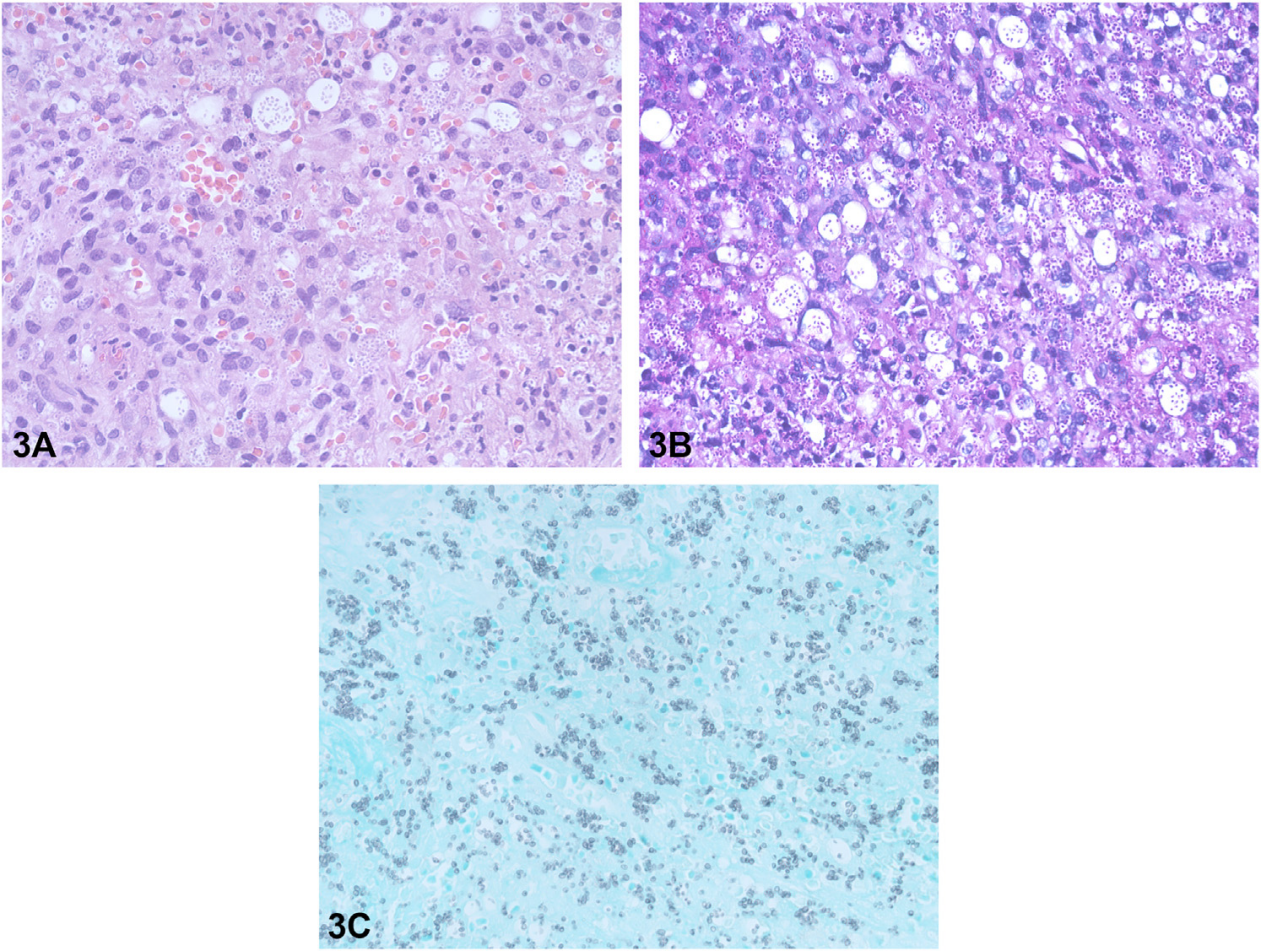

A 50-year-old woman with systemic lupus erythematosus presented with pale painful tongue ulcers and constitutional symptoms for 2 months. Her current medication included prednisolone 5 milligrams per day and mycophenolate mofetil (MMF) 1 to 1.5 grams per day for 2 years. Examination revealed multiple painful ulcers at tip of tongue, lower lip, and painful ulcer deep to muscular layer at dorsal aspect of tongue (Figs 1 and 2). There was no regional lymphadenopathy or hepatosplenomegaly. Serology for HIV is negative. Histopathological, Grocott-Gomori's Methenamine Silver, and Periodic acid-Schiff stain results are shown in Fig 3, *A*-*C*.

**Question 1: What is the most likely diagnosis?**

A. Oral ulcer in systemic lupus erythematosus
B. Herpes simplex virus
C. Histoplasmosis
D. Giant cell arteritis
E. MMF – induced oral ulcer

**Answers:**

A. Oral ulcers in systemic lupus erythematosus – Incorrect. Oral ulcers in systemic lupus erythematous typically present as nonpainful, nonspecific ulcers at palate, buccal mucosa, and fissure at tongue. The histopathological features show vacuolar interface dermatitis with deep, periadnexal, and perivascular infiltration.
B. Herpes simplex virus – Incorrect. The herpes simplex virus lesions typically present as scallop border ulcerations at keratinized mucosa and chronic, enlarging ulcerations involving nonkeratinized mucosa in immunocompromised setting. The histologic findings^1^ are ballooning cells, multinucleated giant cells, and herpetic cytopathic effect including gray margination of the chromatin at the nuclear edge.
C. Histoplasmosis – Correct. Primary oral histoplasmosis is rare. The common oral areas are tongue, palate, buccal mucosa, and gingiva. Hematoxylin and eosin staining reveals 2 to 4 μm budding yeast organisms surrounded by clear space within the cytoplasm of macrophages. The organisms will be highlighted in Grocott-Gomori's Methenamine Silver and Periodic acid-Schiff staining.
D. Giant cell arteritis – Incorrect. Tongue necrosis is an atypical presentation of giant cell arteritis. However, the classic manifestations of giant cell arteritis are headache, jaw claudication, and visual loss. Temporal artery biopsy is the gold standard for diagnosis.^2^
E. MMF – induced oral ulcer – Incorrect. Common presentations are major and minor aphthous ulcers. Histological analyses are nonspecific. The lesions will heal after MMF cessation for weeks.^3^

**Question 2: Tissue fungal culture was negative. Which is the best step in management for this patient?**

A. Urine for antigen detection
B. Peripheral blood smear
C. Molecular-based polymerase chain reaction assay from tissue
D. Blood culture for fungus
E. Excision

**Answers:**

A. Urine for antigen detection – Incorrect. The sensitivity of *Histoplasma* *capsulatum* antigen detection in urine is 92%. A false negative result is presented in granulomatous patients. The sensitivity of penicilliosis is 94.6% to 100% with some false positive results. Urine antigen detection is sensitive in disseminated patients but may be negative in primary cutaneous infection.^4^
B. Peripheral blood smear – Incorrect. In routine peripheral blood smear will sometimes show yeast-like organisms in neutrophils in especially disseminated patients and may not be detected in granulomatous group.^4^
C. Molecular-based polymerase chain reaction assay from tissue – Correct. The molecular-based method is more rapid, has high sensitivity, and is accurate in diagnosis. The sensitivity of the test is 93.6%^5^ to detect fungus in fresh tissue.
D. Blood culture for fungus – Incorrect. The culture will be detected up to 6 weeks after specimen collection. In severe forms of histoplasmosis including disseminated type or pulmonary involvement will have the greatest yield for culture positivity. In milder form will usually remain negative.^4^
E. Excision – Incorrect. There is no evidence of the benefit of surgery in histoplasmosis because histoplasmosis can be cured with medication.

**Question 3: Investigation for disseminated lesion is negative. What is the most appropriate management for this patient?**

A. Intravenous amphotericin B
B. Oral itraconazole
C. Oral voriconazole
D. Oral fluconazole
E. Echinocandins

**Answers:**

A. Intravenous amphotericin B – Incorrect. Intravenous amphotericin B is the drug of choice in severe histoplasmosis and initial treatment. In disseminated patients, mortality rate rises to 83% in nontreated patients, compared with 23% in amphotericin B-treated patients.^1^ In this case, the patient is proved with no evidence of dissemination. Therefore, the antifungal should step down to oral itraconazole.
B. Oral itraconazole – Correct. Itraconazole is the drug of choice in mild to moderate histoplasmosis.^5^
C. Oral voriconazole – Incorrect. Although, voriconazole is a new azole group with the same structure as fluconazole. There are inadequate clinical data to suggest evidence-based recommendation in histoplasmosis treatment. Thus, voriconazole is the second-line alternative to itraconazole.
D. Oral fluconazole – Incorrect. Fluconazole is the second-line treatment because it is less effective compared to itraconazole in both patients with AIDS (63%) and patients without AIDS (50%).^5^
E. Echinocandins – Incorrect. The action of echinocandins is to interfere fungal wall formation by inhibiting ß-(1,3)-D-glucan synthesis. In in vitro study, susceptibility of histoplasmosis to echinocandins group is controversial. The study in infected animal models is ineffective. Consequently, echinocandins are not suggested in Infectious Diseases Society of America guidelines.

## Conflicts of interest

None disclosed.

## References

[1] Silvia V.L., Flávia C.C., Boggio P. (2007). Lupus erythematosus: clinical and histopathological study of oral manifestations and immunohistochemical profile of the inflammatory infiltrate. J Cutan Pathol.

[2] Grant S.W.J., Underhill H.C., Atkin P. (2013). Giant cell arteritis affecting the tongue: a case report and review of the literature. Dent Update.

[3] Plana-Pla A., Solé L.C., Garcia A.B., Valdemoros R.L. (2019). Mycophenolate mofetil-induced mouth ulcers in a kidney transplant patient: case report and literature review. Nefrologia.

[4] Kauffman C.A. (2007). Histoplasmosis: a clinical and laboratory update. Clin Microbiol Rev.

[5] Lau A., Chen S., Sorrell T. (2007). Development and clinical application of a panfungal PCR assay to detect and identify fungal DNA in tissue specimens. J Clin Microbiol.

